# Contrast-enhanced endoscopic ultrasonography for diagnosis of leiomyosarcoma of the inferior vena cava

**DOI:** 10.1055/a-2534-3191

**Published:** 2025-02-20

**Authors:** Wen Zhang, Ming-Yan Cai

**Affiliations:** 1 Department of Gastroenterology, Suzhou Ninth People’s Hospital, Suzhou Ninth Hospital Affiliated to Soochow University, Suzhou, China; 2 Endoscopy Center, Zhongshan Hospital, Fudan University, Shanghai, China

Endoscopic ultrasound (EUS) has become an important diagnostic tool for various diseases 1 . Contrast-enhanced (CE)-EUS has emerged as an effective technique that is complementary to conventional EUS and allows visualization of microvessels and parenchymal perfusion, and more accurate characterization of the lesion 2 .

We report the case of a 46-year-old woman with abdominal pain. On a contrast-enhanced computed tomography scan, a mass of approximately 7 cm in diameter was discovered in the right posterior peritoneum, with compression of the duodenum and inferior vena cava (IVC) ( Fig. 1 ). We decided to perform CE-EUS for this patient ( Video 1 ).

**Fig. 1:**
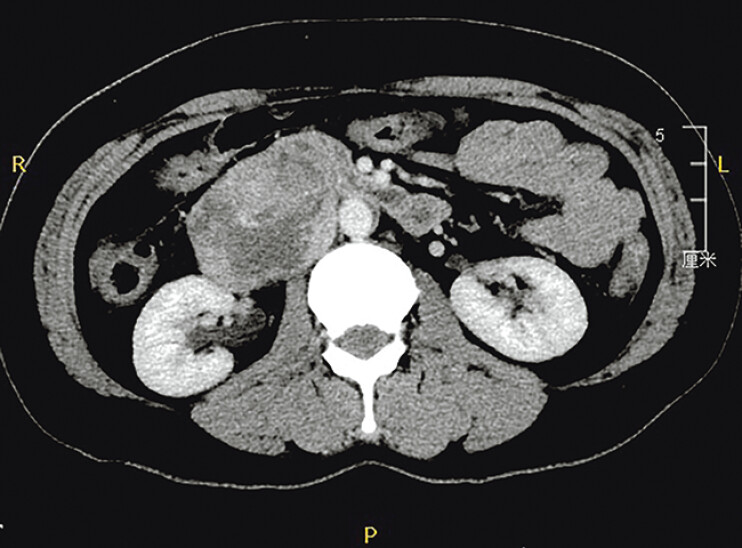
Contrast-enhanced computed tomography scan showed a mass of approximately 7 cm in diameter in the right retroperitoneum.

Contrast-enhanced endoscopic ultrasonography for diagnosis of leiomyosarcoma of the inferior vena cava.Video 1

On CE-EUS, we discovered a solid hypoechoic lesion originating from the IVC wall, independent of the duodenum ( Fig. 2 ). After injection of contrast reagent (Sonovue; Bracco, Milan, Italy), the lesion showed heterogeneous hyper-enhancement into the lesion ( Fig. 3 ). For a pathological diagnosis, EUS-guided fine-needle aspiration (EUS-FNA) of the lesion was performed with a 22-gauge needle (SharkCore; Medtronic, Minneapolis, Minnesota, USA).

**Fig. 2:**
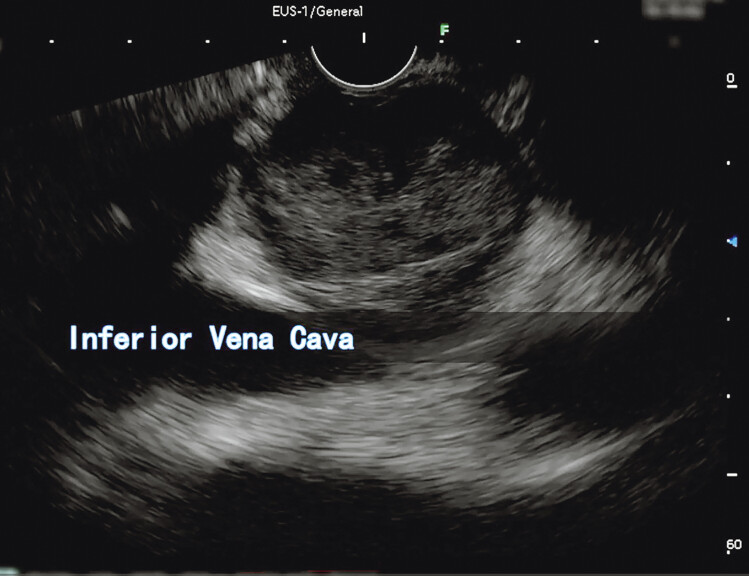
Endoscopic ultrasound showed a 7-cm hypoechoic mass originating from the inferior vena cava.

**Fig. 3:**
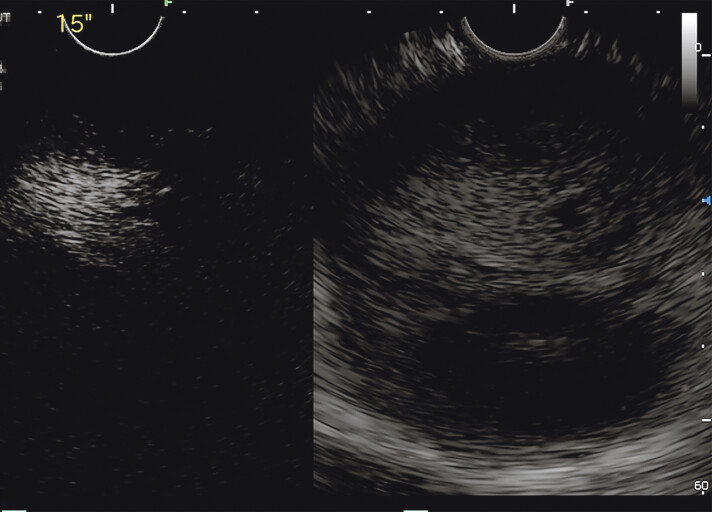
Contrast-enhanced endoscopic ultrasonography showed a heterogeneous hyper-enhancing pattern into the lesion.

Histological examination revealed an abnormal high proliferation of spindle cells, obvious nuclear atypia, and mitotic activity. The immunohistochemical stains revealed positivity for B-cat, EMA, and SMA, and negativity for CD34, CD117, CgA, S100, SOX11, and SYN ( Fig. 4 ). We suspected a malignant spindle cell tumor originating from the IVC, most compatible with leiomyosarcoma. The patient accepted surgical resection and vascular reconstruction.

**Fig. 4:**
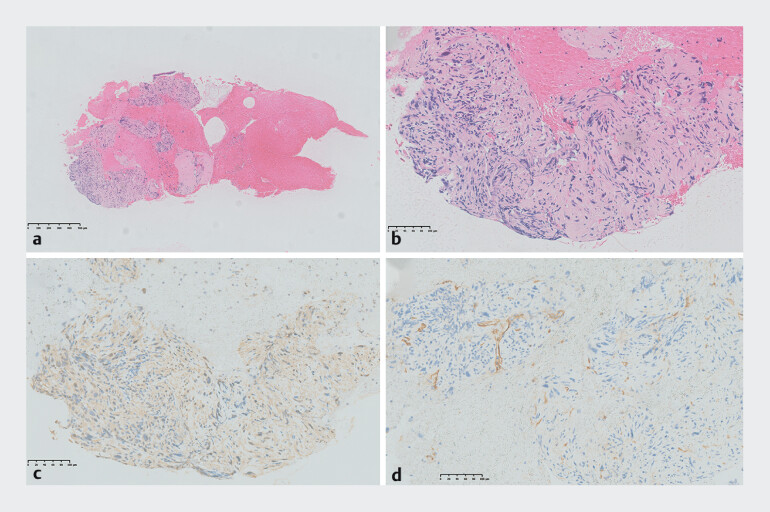
Histopathology of tissue mass samples by endoscopic ultrasound-guided fine-needle aspiration. **a** Original magnification of hematoxylin and eosin stain (×5). **b** Local magnification showed large areas of spindle cells (×200). **c** Tumor cells showed diffuse SMA expression (×200). **d** Tumor cells showed negativity for CD34 (×200).

Finally, the specimen confirmed the diagnosis of leiomyosarcoma. Immunostains showed diffuse positivity for caldesmon, calponin, desmin, EMA, and SMA but negativity for CD10, CD34, CD117, Muc-4, S100, and STAT6.

Primary leiomyosarcoma of the IVC is a rare soft tissue sarcoma 3 . This is the first instance where CE-EUS was applied in the preoperative diagnosis of IVC leiomyosarcoma. The essential role of CE-EUS and EUS-FNA in evaluating IVC leiomyosarcoma preoperatively is remarkable.

Endoscopy_UCTN_Code_CCL_1AF_2AG_3AD

## Notes

Endoscopy E-Videos https://eref.thieme.de/e-videos
*E-Videos* is an open access online section of the journal *Endoscopy* , reporting on interesting cases and new techniques in gastroenterological endoscopy. All papers include a high-quality video and are published with a Creative Commons CC-BY license. Endoscopy E-Videos qualify for HINARI discounts and waivers and eligibility is automatically checked during the submission process. We grant 100% waivers to articles whose corresponding authors are based in Group A countries and 50% waivers to those who are based in Group B countries as classified by Research4Life (see: https://www.research4life.org/access/eligibility/ ). This section has its own submission website at https://mc.manuscriptcentral.com/e-videos .
